# 4547 Understanding the molecular mechanism of natural killer cell deficiency to improve natural killer cell *in vitro* differentiation for therapeutics

**DOI:** 10.1017/cts.2020.101

**Published:** 2020-07-29

**Authors:** Megan Schmit, Ryan Baxley, Emily Mace, Jordan Orange, Jeffery Miller, Anja-Katrin Bielinsky

**Affiliations:** 1 University of Minnesota; 2 Columbia University Irving Medical Center

## Abstract

OBJECTIVES/GOALS: Natural killer (NK) cells are a potential cancer therapeutic but expanding NK cells efficiently *in vitro* is difficult. Natural killer cell deficiency (NKD), a primary immune deficiency affecting only NK cells, is caused by defects in several DNA replication proteins. By studying NKD we will achieve better NK cell *in vitro* differentiation. METHODS/STUDY POPULATION: One patient with NKD has a compound heterozygous mutation in the essential DNA replication protein MCM10. We hypothesize that in individuals with NKD, dramatic telomere erosion from abnormal DNA replication leads to premature senescence and the loss of NK cells. To test our hypothesis, we will knockout one allele of MCM10 or over express *MCM10* in NK cells isolated from blood. We will then monitor telomere length, expansion and cytotoxic activity of these NK cells. To understand the role of MCM10 in early stages of NK cell development we will deplete MCM10 in induced pluripotent stem cells and differentiate these cells into NK cells. During this differentiation we will monitor progression through NK cell developmental stages as well as telomere length and senescence markers. RESULTS/ANTICIPATED RESULTS: Telomeres insulate chromosomes and induce permanent growth arrest (senescence) when they are critically short. We have demonstrated that depletion of a DNA replication protein causes telomere erosion and increases senescence markers. NK cells have shorter telomeres and lower telomerase expression than other immune cells. We predict, this relatively poor telomere maintenance sensitizes NK cells to telomere loss upon depletion of replication proteins. During *in vitro* differentiation, we expect NK cell precursors to undergo premature senescence secondary to telomere shortening. Furthermore, we expect supplementation of DNA replication proteins will enhance NK cell expansion and maturation. DISCUSSION/SIGNIFICANCE OF IMPACT: NKD patients have provided the scientific community with clues as to what proteins NK cells rely on for their development. This project aims not only to understand why these proteins are critical, but to harness that information for cellular anti-cancer therapeutics.

